# Biomarkers of the involvement of mast cells, basophils and eosinophils in asthma and allergic diseases

**DOI:** 10.1186/s40413-016-0094-3

**Published:** 2016-02-11

**Authors:** Dean D. Metcalfe, Ruby Pawankar, Steven J. Ackerman, Cem Akin, Frederic Clayton, Franco H. Falcone, Gerald J. Gleich, Anne-Marie Irani, Mats W. Johansson, Amy D. Klion, Kristin M. Leiferman, Francesca Levi-Schaffer, Gunnar Nilsson, Yoshimichi Okayama, Calman Prussin, John T. Schroeder, Lawrence B. Schwartz, Hans-Uwe Simon, Andrew F. Walls, Massimo Triggiani

**Affiliations:** Laboratory of Allergic Diseases, National Institute of Allergy and Infectious Diseases, National Institutes of Health, Bethesda, MD 20892 USA; Division of Allergy, Department of Pediatrics, Nippon Medical School, Tokyo, Japan; Department of Biochemistry and Molecular Genetics, College of Medicine, University of Illinois, Chicago, IL USA; Harvard Medical School, Brigham and Women’s Hospital, Boston, MA USA; Department of Pathology, University of Utah Health Sciences Center, Salt Lake City, UT USA; The School of Pharmacy, University of Nottingham, Nottingham, United Kingdom; Department of Dermatology, University of Utah, School of Medicine, Salt Lake City, UT USA; Virginia Commonwealth University, Children’s Hospital of Richmond, Richmond, VA USA; Department of Biomolecular Chemistry, University of Wisconsin, Madison, WI USA; Human Eosinophil Section, Laboratory of Parasitic Diseases, National Institute of Allergy and Infectious Diseases, National Institutes of Health, Bethesda, MD USA; University of Utah, Salt Lake City, UT USA; The Hebrew University of Jerusalem, Jerusalem, Israel; Clinical Immunology and Allergy, Department of Medicine, Karolinska Institute and Karolinska University Hospital, Stockholm, Sweden; Allergy and Immunology Group, Research Institute of Medical Science, Nihon University Graduate School of Medicine, Tokyo, Japan; Division of Allergy and Clinical Immunology, Department of Medicine, Johns Hopkins University School of Medicine, Baltimore, MD USA; Virginia Commonwealth University, Richmond, VA USA; University of Bern, Institute of Pharmacology, Bern, Switzerland; Southampton General Hospital, Immunopharmacology Group, Southampton, Hampshire UK; Division of Allergy and Clinical Immunology, University of Salerno, Salerno, Italy

## Abstract

Biomarkers of disease activity have come into wide use in the study of mechanisms of human disease and in clinical medicine to both diagnose and predict disease course; as well as to monitor response to therapeutic intervention. Here we review biomarkers of the involvement of mast cells, basophils, and eosinophils in human allergic inflammation. Included are surface markers of cell activation as well as specific products of these inflammatory cells that implicate specific cell types in the inflammatory process and are of possible value in clinical research as well as within decisions made in the practice of allergy-immunology.

## Introduction

Eosinophils, mast cells and basophils are major effector cells of innate immunity and play a fundamental role in the mechanisms of defense against bacterial, viral and parasitic infections. These cells are distinct in terms of development, maturation and location within tissues. Eosinophils and basophils fully mature in the bone marrow and under physiological circumstances circulate as blood cells. Mast cells leave the bone marrow as progenitors and reach their final maturation within peripheral tissues. However, these three types of cells are often involved together in a variety of pathological conditions when they are activated and accumulate in inflamed tissues where they release a wide spectrum of chemical mediators of inflammation.

Given their strategic location at the major body surfaces including the skin and the lining of the lung and gastrointestinal tract, mast cells are among the first cells to recognize danger signals coming from the external environment. Once activated, mast cells help orchestrate the early phases of the innate immune response, promote the recruitment of other inflammatory cells and participate in the initiation and regulation of adaptive immunity. Eosinophils and basophils are recruited within organs and tissues following inflammatory and chemotactic stimuli that are produced by inflammation. Mast cells, eosinophils and basophils are therefore activated simultaneously in many diseases including infections, allergic and autoimmune disorders and cancer. Investigation of the role and function of mast cells, eosinophils and basophils in human disease has been hampered by difficulties in obtaining cells from human sources in sufficient number to perform suitable in vitro studies. Mature human mast cells can be retrieved only from tissues whereas eosinophils and basophils circulate in the blood in low numbers. Fortunately, new and efficient methods to purify these cells from human blood and tissues have been developed and now allow reproducible studies to explore their role in various pathophysiological conditions. At the same time, highly sensitive techniques are now available to measure mediators and to detect cell surface molecules, even from limited number of cells. These major improvements in cell purification, flow cytometry and mediator measurement have contributed to the identification of molecules that are either released from or expressed on the surface of activated mast cells, eosinophils and basophils and have been associated with involvement of these cells in human disease. In some cases, these molecules have provided important clues into the mechanisms by which mast cells, eosinophils and basophils participate in the development of human disease.

## Eosinophils

### Surface markers of activation

Recruitment of eosinophils from the circulation to tissues in disease requires that eosinophils become activated [1–3]. Up-regulation, and in some cases down-regulation, of surface proteins on blood eosinophils and activated conformations of integrins or Fc receptors have been associated with aspects of allergic disease (Table 1) [1–4]. Up-regulation or down-regulation in the table refers to altered cell-surface protein expression regardless of the mechanism, which may be mobilization from intracellular stores or the result of altered transcription or translation and may mean a change in average level on all cells or a change in the proportion of positive cells [1]. Some studies are on purified eosinophils, whereas some are on unfractionated blood cells. The latter has advantages, including the requirement for only a small amount of blood.

**Table 1:** Eosinophil surface proteins associated with allergic disease or aspects of allergic disease

Protein	Observation	Reference
CD9	Up-regulated during allergy season	[1]
CD40	Correlates inversely with esophagus eosinophilia in EoE	[6]
CD44	Higher in well-controlled than in poorly controlled asthma	[1]
	Down-regulated in EoE	[5]
CD45RO	Up-regulated in mild-moderate asthma	[1]
CD48	Up-regulated in allergic asthma	[1]
CD66b (CEACAM8)	Up-regulated in untreated EoE	[133]
CD69	Up-regulated in severe atopic dermatitis	[10]
CD137 (TNFRSF9, ILA, 4-1BB)	Up-regulated in atopic dermatitis and allergic asthma	[134]
α_L_ integrin (CD11a)	Up-regulated in allergic asthma	[1]
α_M_ integrin (CD11b)	Up-regulated during allergy season	[1]
	Correlates inversely with PC_20_	
α_X_ integrin (CD11c)	Up-regulated in EoE	[5]
β_2_ integrin (CD18)	Correlates with esophagus eosinophilia in EoE	[6]
CCR3	Down-regulated in EoE	[5]
	Correlates inversely with esophagus eosinophilia in EoE	[6]
CRTH2 (Prostaglandin D2 R2, CD294)	Up-regulated in EoE	[5]
FcαRI (CD89)	Up-regulated in allergic rhinitis or allergic asthma	[1]
FcεRII (CD23)	Up-regulated in EoE	[5]
FcγRIII (CD16)	Up-regulated in allergic rhinitis or allergic asthma	[1]
Galectin-3	Up-regulated in allergy	[1]
ICAM-1 (CD54)	Up-regulated in EoE	[5]
IL-2Rα (CD25)	Up-regulated in some patients with asthma	[7]
IL-17RA (subunit of IL- 25R)	Up-regulated in mild allergic asthma	[1]
IL-17RB (subunit of IL- 25R)	Up-regulated in mild allergic asthma	[1]
Neuropeptide S receptor	Up-regulated in severe asthma	[1]
Activated β_1_ integrin (CD29)	Intermediate-activity state (recognized by mAb N29) increased in non-severe but not severe asthma	[1]
	Correlates inversely with FEV_1_ after or during ICS withdrawal in mild asthma and predicts decreased FEV_1_ in ROC curve analysis	
	Correlates with FENO after ICS withdrawal in mild asthma	
	Correlates inversely with FEV_1_/FVC in younger subjects with non-severe asthma	
	Correlates inversely with FEV_1_/FVC in phenotype cluster 1 (mild atopic asthma).	
	At 48 h after segmental lung antigen challenge, correlates with late-phase FEV_1_ fall after whole-lung antigen challenge in mild allergic asthma	
Activated β_2_ integrin (CD18)	Intermediate-activity state (recognized by mAb KIM-127) correlates with BAL eosinophilia in mild allergic asthma	[1]
Activated FcγRII (CD32)	Activated state (recognized by mAb A17 or A27) increased in mild asthma	[1]
	Correlates with FENO in asthma	

Note: Observations refer to expression level, usually determined by flow cytometry, and are on blood eosinophils. For more original references, please see reference list in [1]

Several proteins, including CD48, CD137, CRTH2, ICAM-1, IL-25R, integrins, and Fc receptors have been reported to be up- or down-regulated in one or more diseases when compared to normal, non-allergic healthy individuals (Table 1). At least some changes appear disease-specific. α_X_ integrin, CRTH2, FcεRII (CD23), and ICAM-1 are up-regulated on eosinophils in eosinophilic eosophagitis (EoE) but not in airway allergies like atopic asthma or allergic rhinitis [5]; IL-25R is up-regulated in mild allergic asthma but not in atopic non-asthmatic subjects [1]; CD44 and CCR3 are down-regulated in EoE but not in airway allergies [5]. There are conflicting reports in the case of some proteins. Some authors found FcγRIII (CD16) to be up-regulated in airway allergies [1], whereas others [5] found no change compared to normal healthy individuals.

Expression or activation of some proteins has been found to be associated with clinical findings including extent of disease (Table 1). β_2_ integrin positively correlates while CD40 and CCR3 inversely correlate with tissue eosinophilia in EoE [6]. Activated β_1_ integrin, specifically the number of β_1_ integrins in the intermediate-activity conformation which is recognized by mAb N29, is significantly increased in mild to moderate (less severe) asthma compared to healthy donors, but is not significantly increased in severe asthma [1, 2]. Expression of the N29 epitope correlates inversely with lung function in non-severe asthma, i.e., higher N29 reactivity is associated with lower pulmonary function [1, 2]. In addition, by ROC curve analysis, N29 reactivity predicts decreased lung function in non-severe asthma [1, 2]. Partially activated β_2_ integrin correlates with broncheoalveolar lavage (BAL) eosinophilia in patients with mild allergic asthma [1, 2]. CD25 can be upregulated by IL-5 and GM-CSF in vitro and its expression is increased on BAL eosinophils in eosinophilic asthma and tissue eosinophils in atopic dermatitis and EoE [7, 8]. Moreover, HLA-DR expression was observed on sputum eosinophils of patients with asthma [9]. CD69 and neuropeptide S receptor are up-regulated on blood eosinophils in severe disease compared to non-severe disease (Table 1) [1, 10]. In contrast, CD44 and activated β_1_ integrin are increased in well-controlled/non-severe, but not in poorly controlled/severe, asthma. This is suggested to be due to continuous extravasation of the high-expressing eosinophils in severe disease [1].

The expression of some proteins has been found to change in response to intervention, such as administration of corticosteroids or an anti-IL-5 antibody (Table 2) [1, 6, 7, 11]. Specifically, the expression of β_2_ integrin is decreased by corticosteroids and CCR3 is decreased by anti-IL-5 (in EoE) [6, 11]. Anti-IL-5 causes decreased β_2_ activation and, after segmental lung antigen challenge, decreased levels of α_L_, α_M_, and β_2_ integrins as well as PSGL-1 (in mild allergic asthma) [1]. The utility of these potential markers, e.g., as predictors of aspects of allergic disease or of response to intervention, and differences and similarities in the behavior of markers among different allergic diseases, need to be explored further in clinical studies.

**Table 2:** Eosinophil surface proteins reported to respond to intervention

Protein	Observation	Reference
α_L_ integrin (CD11a)	Decreased by anti-IL-5 (mepolizumab) after segmental lung antigen challenge (in mild allergic asthma)	[1]
α_M_ integrin (CD11b)	Decreased by anti-IL-5 (mepolizumab) after segmental lung antigen challenge (in mild allergic asthma)	[1]
β_2_ integrin (CD18)	Decreased by anti-IL-5 (mepolizumab) after segmental lung antigen challenge (in mild allergic asthma)	[1]
	Decreased by topical corticosteroids (in EoE)	[6]
CCR3 (CD193)	Decreased by anti-IL-5 (mepolizumab) (in EoE)	[11]
IL-2Rα (CD25)	Decreased by corticosteroid and other anti-inflammatory drug treatment	[7]
IL-5Rα (CD125)	Increased by anti-IL-5 (mepolizumab)	[1]
PSGL-1 (CD162)	Decreased by anti-IL-5 (mepolizumab) after segmental lung antigen challenge (in mild allergic asthma)	[1]
Activated β_2_ integrin (CD18)	Intermediate state (recognized by mAb KIM-127) decreased by anti-IL-5 (mepolizumab) (in mild allergic asthma)	[1]

Note: Observations refer to expression level, usually determined by flow cytometry, and are on blood eosinophils

### Major basic protein

Major basic protein (MBP)-1 and MBP-2 are two of the five basic proteins found within secondary eosinophil granules, the others being eosinophil-derived neurotoxin (EDN) also known as ribonuclease (RNase) 2, eosinophil cationic protein (ECP) also known as RNase3, and eosinophil peroxidase (EPX) (Table 3) [12]. MBP comprises the crystalloid core of the specific eosinophil granule, accounting for a major portion of the eosinophil granule protein, and has a high isoelectric point, calculated at greater than pH 11, so strongly basic that it cannot be measured accurately [13]. It is now known that MBP is expressed as two homologs, MBP-1 and MBP-2, coded by different genes on chromosome 11. MBP-1 and presumably MBP-2, is translated as a proprotein with a strongly acidic pro-portion [14]. The combination of the pro-section with MBP-1 or MBP-2 yields a molecule with a relatively neutral pI, 6.2. ProMBP-1 lacks the cytotoxic properties of MBP-1 and MBP2. Synthesis as a neutral pro-protein may function to protect the cell during transport from the Golgi apparatus to the granule. ProMBP-1 is the predominant form of MBP-1 in the blood of pregnant women and circulates as a complex with pregnancy-associated plasma protein-A (PAPP-A), with angiotensin, and with complement component, C3dg. It localizes to placental X cells and functions as a novel enzyme inhibitor of PAPP-A to modify its ability to release active insulin-like growth factor during primate pregnancy.

**Table 3:** Biologic activities of human eosinophil granule proteins

Granule protein	Biologic activities
Major Basic Protein (MBP)	Helminthotoxin and cytotoxin; bactericidal, platelet agonist; causes histamine release from basophils and rat mast cells; neutralizes heparin; increases bronchial reactivity to methacholine; provokes bronchospasm; activates neutrophils
Major Basic Protein-2 (MBP-2)	Cytotoxin; causes histamine release and LTC_4_ release from basophils; causes superoxide and IL-8 release from neutrophils
Eosinophil Cationic Protein (ECP)	Helminthotoxin, neurotoxin, weak RNase activity, bactericidal, virucidal; inhibits cultures of peripheral blood lymphocytes; causes histamine release from rat mast cells; neutralizes heparin, alters fibrinolysis
Eosinophil –Derived Neurotoxin (EDN)	Neurotoxin, RNase activity, virucidal, weak helminthotoxin; inhibits cultures of peripheral blood lymphocytes; same as eosinophil protein X
Eosinophil Peroxidase (EPX)	In presence of H_2_O_2_ plus halide: kills microorganisms and tumor cells; causes histamine release and degranulation from rat mast cells; inactivates leukotrienes in absence of H_2_O_2_ plus halide; kills *Brugia* microfilariae; damages respiratory epithelium; provokes bronchospasm in primates. Generates reactive oxidants and free radicals

Both MBP-1 and MBP-2 are present in eosinophil granules [15]; MBP-1 is also present in basophils in a much lesser concentration than in eosinophils, but MBP-2 is not present in basophils. Mature eosinophils lose their ability to transcribe mRNA encoding MBP-1, indicating that all MBP-1 stored in the crystalloid granule cores is synthesized during early eosinophil development. MBP-2 is approximately 100 times less basic than MBP-1, with a calculated pI of 8.7. MBP-1 and MBP-2 have 42 identical amino acids of the approximately 117 in each of these proteins. The crystal structure of MBP-1 indicates that it is a member of the C-type lectin family. Comparative analyses of the biological effects of MBP-1 and MBP-2 demonstrate that they are similar in cytotoxic and cytostimulatory effects, but with reduced potency of MBP-2. Most of the eosinophil granule protein activities have been characterized for MBP-1. MBP-1 directly damages helminths and bacteria, and lethally damages mammalian cells and tissues. MBP-1 and MBP-2 stimulate histamine and LTC 4 release from human basophils and MBP-1 activates primed mast cells. Both MBP-1 and MBP-2 stimulate neutrophils. MBP-1 is a potent platelet agonist, causing release of 5-hydroxytryptamine and promoting clotting. MBP-1 is a potent vasodilator and increases vascular permeability. MBP-1 is elevated in the sputum of patients with asthma and is deposited in cardiac and skin lesions of patients with hypereosinophilic syndromes. Recently it has been shown that the toxicity of MBP-1 is regulated by aggregation [16].

### Eosinophil Cationic Protein (ECP)

ECP occurs as a single chain protein with apparent molecular masses ranging from 15.7 to 22 kDa largely due to glycosylation and is stored in the granules as a highly glycosylated, non-toxic protein. ECP is expressed by eosinophils and is present only in humans and primates. Upon eosinophil degranulation, ECP is deglycosylated and acquires cytotoxic capabilities [17].

Among the four eosinophil basic proteins, ECP is the most widely used marker, to monitor diseases involving eosinophils and can be measured in serum/plasma and BAL. The majority of reports indicate that ECP levels provide useful information allowing not only monitoring of the disease course, but also treatment stratification [18, 19]. Levels of ECP in serum are consistently higher than in plasma, since eosinophils release ECP during the coagulation process, which is both time- and temperature- dependent [20]. Compared to eosinophil numbers, serum ECP levels decline with a delay following anti-IL-5 antibody therapy in patients with eosinophilia [21], pointing to the possibility that released ECP may remain in the circulation longer than the cellular source.

The levels of ECP in plasma likely reflect the ECP concentrations in blood at the time of sampling. Therefore, plasma ECP levels may better reflect the intensity of eosinophil activation than serum ECP levels, but no data are available that would support this assumption. ECP concentrations in other body fluids also likely reflect the activation stage of eosinophils. Taken together, the levels in serum/plasma and/or other body fluids largely correlate with the intensity of eosinophilic inflammation that is characterized by both numbers and activation of eosinophils. It should be noted, however, that ECP has been reported to be present in neutrophils which should be taken into consideration [22].

### Eosinophil Peroxidase (EPX)

EPX is an eosinophil-specific cationic protein that is localized in the matrix of the secondary granules. Synthesized as a single chain precursor, EPX is proteolytically processed into a heavy chain (50–57 kDa) and a light chain (11–15 kDa). Although EPX has 68 % sequence identity to the neutrophil myeloperoxidase (MPO), patients with MPO deficiency have normal levels of EPX, consistent with differing mechanisms of expression of the two proteins.

In the presence of hydrogen peroxide, EPX catalyzes the oxidation of halides, pseudohalides and nitric oxide to form hypohalous acids, nitric dioxide and other reactive species. These products have been implicated in both inflammatory and anti-inflammatory processes including killing of pathogens [23], regulation of cholinergic nerve plasticity and inactivation of leukotrienes [24]. EPX has also been shown to be a useful biomarker of eosinophilic inflammation in a wide variety of clinical samples. Intracellularly, EPX mediates post-translational tyrosine nitration of all of the eosinophil granule proteins, including itself, during eosinophil maturation [25]. Despite these seemingly important functions, genetic deficiency in EPX has been described in humans and does not appear to be associated with a clinical phenotype.

### Charcot-Leyden crystal protein

Charcot-Leyden Crystal Protein (CLC/Galectin-10) forms hexagonal bipyramidal crystals, hallmarks of eosinophil participation in allergic inflammatory reactions, found in tissues, body fluids and secretions. CLC, a 142 amino acid protein of 16.5 kDa [26], was initially identified as eosinophil lysophospholipase [27], but is now assigned to the galectin superfamily (Galectin-10) [28, 29]. Importantly, CLC/Gal-10 lacks lysophospholipase activity, and the weak enzymatic activity initially attributed to CLC was due to contamination by other lysophospholipases expressed by eosinophils [30]. In addition to eosinophils and basophils [29], CLC/Gal-10 is expressed by human regulatory T cells (Tregs) [31]. Proteomics of CD4 + CD25+ Tregs identified CLC/Gal-10 as a novel biomarker and essential for Treg anergy and suppressive functions on T cell activation [31]. CLC/Gal-10 is a potentially useful biomarker of eosinophil/basophil involvement in asthma, allergic rhinitis and other eosinophil-associated inflammatory diseases [32]. CLC/Gal-10 levels measured by ELISA are a sensitive and specific biomarker of active eosinophilic inflammation in EoE [33], differentiating patients with active EoE from treated patients in remission and those with gastroesophageal reflux disease, and is highly correlated with the numbers of tissue eosinophils. CLC/Gal-10 is also a potential biomarker for eosinophilic airway inflammation in induced sputum [34]. In celiac disease, CLC/Gal-10 expression is related to disease activity and tissue eosinophils in intestinal lesions [35]. Genetic variations (single nucleotide polymorphisms [SNPs]) in the promoter region of the *CLC* gene identify potential susceptibility biomarkers for allergic rhinitis [36], with variation compatible with recessive inheritance and increased levels of CLC/Gal-10 in nasal fluid of patients with allergic rhinitis during allergy season.

### Eosinophil-derived neurotoxin (EDN, RNase2)

EDN, a small cationic protein belonging to the ribonuclease A (RNase A) superfamily, is localized to the matrix of the eosinophil’s large secondary granule, and is also known as RNase-2, non-secretory RNase, and RNase-U_s_, the latter based on its specificity toward uridine-containing nucleotides. EDN has been isolated from a wide variety of sources including eosinophils, placenta, liver, and urine. There is ~3.3 μg of EDN/10^6^ eosinophils, but it is not specific to eosinophils, being expressed in smaller amounts by both basophils and neutrophils [37]. EDN was first identified based on its induction of a paralytic syndrome of ataxia, incoordination, and spasmodic paralysis when injected intrathecally into rabbits [38]. The RNase activity of EDN is a prerequisite for its cytotoxic, neurotoxic and antiviral activities [39, 40]. Unlike other eosinophil granule proteins, EDN is a poor cationic toxin with limited toxicity for helminth parasites and mammalian cells, but as a ribonuclease, it is considerably more effective against single-stranded RNA viruses [39]. EDN activates human dendritic cells (DCs) [41], induces phenotypic and functional maturation of DCs, and acts as an alarmin that activates the TLR2-MyD88 signaling pathway [42]. As a measure of eosinophil activation in vitro, EDN has been a biomarker of choice, since eosinophils have been shown to secrete >80 % of available EDN compared to the other granule cationic proteins. As a biomarker of eosinophil-associated diseases, EDN levels are significantly increased in blood (serum/plasma), urine and other body fluids, and tissue biopsies and esophageal luminal secretions [33]. EDN levels are significantly increased in the blood of patients with Onchocerciasis during an eosinopenia induced by treatment with diethylcarbamazine [43]. In EoE, plasma EDN levels are significantly elevated in active EoE, with moderately good sensitivity and specificity for differentiating EoE from patients with GERD and normal subjects, but only when combined with absolute blood eosinophil counts [44, 45]. Studies comparing EDN levels in esophageal biopsy extracts with the minimally invasive Esophageal String Test (EST), show elevated levels of EDN that distinguish patients with active EoE from those in treatment-induced remission, GERD and normal esophagus [33]. Serum EDN levels measured 3 months post-respiratory syncytial virus (RSV)-induced bronchiolitis may be a predictive biomarker of recurrent wheezing in infants [46]. In summary, for already diagnosed eosinophil-associated inflammatory diseases, e.g., EoE, EDN may have utility as a blood and/or tissue biomarker for following patient responses to treatment; however, EDN’s value may be limited by its expression in neutrophils, e.g., for distinguishing eosinophilic vs. neutrophilic phenotypes of asthma using induced sputum.

## Mast cells

### Histamine

Histamine is the main biogenic amine released upon IgE-receptor activation by human mast cells (3–8 pg/cell) and basophils [47]. Histamine measured in blood in association with an allergic reaction cannot thus be considered a specific marker of mast cell activation. Histamine is present in substantial amounts in many fermented foods and beverages, and this must be taken into consideration when measuring histamine levels. Histamine can be measured in several body fluids, and been shown to be increased in bronchoalveolar lavage fluid from patients with allergic asthma and plasma from patients with atopic dermatitis or chronic urticaria [48, 49]. Measurement of plasma histamine is problematic due to the short half-life of histamine (10–30 min) [50]. Histamine is rapidly metabolized either by methylation into methyl histamine catalyzed by histamine N-methyltransferase, or oxidative deamination into imidazole acetaldehyde catalyzed by diamine oxidase. The metabolite, 1-methyl-4-imidazole acetic acid (tele-MIAA), represents 70–80 % of metabolized histamine [51] and is excreted in urine. Determination of levels of histamine metabolites in urine provides a non-invasive means by which to monitor global histamine turnover in humans. Such an analysis is usually performed on urine collected over 24 h, but shorter collection times and even spot samples may be used. Increased levels of histamine metabolites in the urine can be sign of systemic mastocytosis and/or mast cell and basophil activation [52]. There are several methods available to measure histamine, including ELISA and solid phases, e.g., glass fibers. These are primarily useful for in vitro measurement of histamine released from mast cells and basophils. Histamine metabolites in the urine may also be measured by high-performance liquid chromatography (HPLC) and HPLC coupled to mass spectrometry (LC – MS) [51, 53].

### Heparin

Heparin is produced by mast cells and human lung mast cells contain approximately 2.4 to 7.8 micrograms of heparin per 10^6^ cells [54]. Human heparin is associated with the collections of mast cells associated with urticaria pigmentosa (maculopapular lesions) [55]. In rare cases of advanced systemic mastocytosis, a heparin-like anticoagulant may be released which leads to hemorrhagic complications [56]. However, in most cases, the thrombin time and partial thromboplastin time remains normal in those with mast cell proliferative disorders. Basal endogenous heparin levels in blood have been reported to be above the laboratory-specific range in approximately 40 % of patients classified as having mast cell activation disease [57]. It has also been suggested that heparin-initiated bradykinin formation plays a role in mast cell-mediated diseases [58]. Thus measurement of mast cell-derived heparin should be considered in mast cell mediated diseases where there is clinical evidence of hemorrhagic complications. However, in the vast majority of mast cell mediated inflammatory reactions, blood levels of heparin will remain within the normal range.

### Tryptase

The human tryptase locus on chromosome 16 includes genes that encode α- and β- tryptases [59], which are major protein products of human mast cells, much lesser amounts being expressed by basophils [60]. α/β-Protryptases are processed to maturity by cathepsins B and L, while β-protryptase also can be sequentially processed by autocatalysis and cathepsin C [61, 62]. Mature β-tryptase is proteolytically active as a homotetramer, whereas mature α-tryptase is not, despite being 93 % identical in amino acid sequence. Protryptases are constitutively secreted by resting mast cells, whereas mature tryptases, which are stored in secretory granules, are only secreted by activated mast cells. Baseline serum levels of α/β-tryptases (pro + mature), ranging from 1–11 ng/ml in healthy subjects, reflecting primarily their genetic background [63], serve as a minor diagnostic criterion for systemic mastocytosis when >20 ng/ml (Table 4). However, in subjects with systemic anaphylaxis to insect stings, serum basal tryptase levels in the upper normal range [64] or when between 11 and 20 [65], also raise suspicion for an underlying clonal mast cell disorder. Acute serum tryptase levels, collected within 4 h of a putative anaphylactic event, if increased > (2 + 1.2*baseline level), provide laboratory evidence for mast cell activation [66].

**Table 4:** Tryptase levels in relation to mastocytosis amd anaphylaxis

Condition	Serum total tryptase level (ng/ml)	Comments
Healthy	1–11	Level is primarily genetically determined
Mastocytosis	>20	Levels <20 do not rule out mastocytosis, particularly in patients who have had systemic anaphylaxis to an insect sting
Anaphylaxis	>[2 + (1.2*baseline level)]	Acute serum within 4 h of event onset; baseline serum either before acute event or >24 h after all signs and symptoms resolve

### Chymase

Human chymase is a chymotrypsin-like serine protease specific for mast cells. It is found in a subset of human mast cells, usually in conjunction with human mast cell carboxypeptidase A3. It is released from mast cells in large complexes containing heparin proteoglycan and carboxypeptidase and distinct from complexes containing tryptase [67]. Human chymase is the major non-angiotensin-converting enzyme (ACE) that generates angiotensin II as well as a non-endothelin converting-enzyme (ECE) that generates endothelin-1. As such, chymase is thought to participate in multiple inflammatory responses in the vasculature, including blood pressure regulation and plaque instability [68]. Chymase degrades lipoproteins which promotes macrophage foam cell formation. Chymase can also degrade extracellular matrix, generate fibronectin and transforming growth factor-β and activate IL-1β, and has been implicated in the pathogenesis of tissue fibrosis and wound healing [69, 70]. In human serum, chymase is subject to inhibition by endogenous circulating inhibitors, including α-1 antitrypsin, α-1 antichymotrypsin, α-2 macroglobulin and locally secreted inhibitors including secretory leukocyte protease inhibitor (SLPI) [71]. An immunoassay for the detection of released chymase in biologic fluids is not currently available. Raymond et al. developed an α-2 macroglobulin capture assay using a synthetic substrate which detects enzymatic activity in chymase-spiked serum with a threshold of approximately 30 pg/ml, and reveals detectable chymase activity in serum of most subjects with systemic mastocytosis [72].

### Carboxypeptidase A3

Carboxypeptidase was first identified in human mast cells in 1989, with enzyme activity varying from 0.5 to 16 ug per 10^6^ mast cells depending on the mast cell population [73]. Mast cells containing Carboxypeptidase A3 (CA3) have been reported in association with allergic disease of both the lower and upper airways [74, 75]. CPA3 is also one of 10 genes overexpressed in the bone marrow mononuclear cells of adult patients with systemic mastocytosis [76].

Plasma levels of mast cell carboxypeptidase have been reported to be elevated in children with allergic diseases compared to normal children (1.2+/− 0.8 vs 0.06 +/− 0.5 ng/ml) [77]. Serum CPA3 levels have also been reported to be elevated in those with a clinical diagnosis of anaphylaxis but not in the serum of adult healthy blood donors or individuals with a diagnosis of asthma of another IgE-mediated allergic disease. The serum levels of tryptase and of CPA3 after anaphylaxis do not necessarily correlate [78]. CPA3 levels appear to remain elevated longer than the tryptase levels. Further, CPA3 serum levels have also been reported to be detected in individuals with anaphylaxis where elevations in total serum tryptase levels were not observed.

### Prostaglandin D2 and cysteinyl leukotrienes

Prostaglandin D2 (PGD2) and cysteinyl leukotrienes (Cyst LTs) are the major lipid mediators synthesized after mast cell activation [79]. They are released shortly after the granule contents as part of the immediate mast cell response. Prostaglandins and leukotrienes are synthesized from arachidonic acid (AA) which is released by the action of cytosolic phospholipase A2 on membrane phospholipids [80].

In the PG pathway, AA is first converted to PGG2 by cycloxygenase (COX)-1 and COX-2, and then reduced to PGH2. The latter serves as the precursor for PGD2 as well as other prostanoids PGE2, PGF2a, PGI2, and thromboxane A2 through terminal PG synthases. PGD2 is also produced by eosinophils and in lesser quantities by other immune cells such as Th2 cells and dendritic cells. Non-hematopoietic tissues such as brain, heart, lungs and kidneys can also produce PGD2 via lipocalin type PGD2 synthase. PGD2 exerts its biologic actions by binding to two receptors named DP1 and DP2. End organ functions of PGD2 in humans include vasodilation and bronchoconstriction. Pulmonary, nasal and ocular allergen challenges result in increased levels of PGD2 in relevant biologic fluids. PGD2 and its metabolite 11b-PGF2a are found increased in the urine of patients with mastocytosis and mast cell activation syndrome [81, 82]. The diagnostic and therapeutic clinical utility of PGD2 as a marker of mast cell activation has been limited by its production by cells other than mast cells.

Historically known as the slow reacting substance of anaphylaxis [83], LTC4, LTD4 and LTE4 are collectively termed as CysLTs. 5 lipoxygenase (5-LO) along with the perinuclear membrane protein called 5-LO activating protein convert AA to 5-hydroxyperoxyeicosotetraenoic acid which then gets dehydrated to the unstable leukotriene precursor LTA4. LTA4 is then conjugated to reduced gluthatione by LTC4 synthase, which is secreted out of the cells. LTC4 is converted first to LTD4, then to the most stable form LTE4 extracellularly. CysLTs are generated by mast cells, basophils, eosinophils, macrophages and myeloid dendritic cells. There are at least 3 CysLT receptors: CysLT1R, CysLT2R and CysLT3R (GPR99) [84]. As a mediator associated with allergic inflammation, LTC4’s biological importance resides in its capacity to induce smooth muscle contraction at a concentration that is on the order of 100–6000 times lower than for histamine, and this contraction also lasts substantially longer when initiated with LTC4 than with histamine. CysLTs also induce wheal and flare reactions (increased vascular permeability) in humans. Urinary LTE4 was found to be elevated in systemic mastocytosis and correlated with 24 h urine N-methylhistamine and serum tryptase levels [85].

### CD25, CD63, CD203c, FcεR1

The stem cell factor receptor KIT (CD117) is expressed on on mast cells independent of tissue site, maturation or activation status. In systemic mastocytosis, KIT is often expressed in mast cells in a mutated and constitutively activated form. CD63 and CD203c expression are upregulated following aggregation of FcεRI, and they are overexpressed on neoplastic mast cells in systemic mastocytosis [86]. Mast cells in systemic mastocytosis express CD30 and CD25.

CD63 belongs to a family of tetraspanins, which comprise a superfamily of cell surface-associated membrane proteins characterized by four transmembrane domains [87]. At the cell surface, tetraspanins form networks with a diversity of proteins, including cell surface receptors (MHCII, CD3, FcεRI, CXCR4), kinases (phosphatidylinositol 4-kinase and the Src family tyrosine kinases Lyn and Hck), integrins (α4β1, α3β1, α6β1, LFA-1 and β2), and other tetraspanins (CD81, CD82, CD9, CD151) [87]. The networks of tetraspanins with proteins are called tetraspanin-enriched microdomains. CD63 is also abundantly present in late endosomes and lysosomes [87]. CD63 at the cell surface is endocytosed via a clathrin-dependent pathway. In late endosomes, CD63 is enriched on the intraluminal vesicles, which are secreted by specialized cells as exosomes through fusion of endosomes with the plasma membrane [87]. CD63 has been identified as an activation marker for basophils and mast cells [88]. CD63 expression is rapidly upregulated following allergen challenge and reaches a maximum after 20–30 min [89]. CD63 is also expressed on neutrophils, dendritic cells, T cells and eosinophils [88]. Anti-CD63 antibodies inhibit FcεRI-induced mast cell degranulation in vitro and FcεRI-induced allergic reaction in vivo [90, 91].

CD203c (E-NPP3) belongs to a family of ecto-nucleotide pyrophosphates/phosphodiesterases (E-NPPs). E-NPPs catalyze the cleavage of phosphodiester and phosphosulfate bonds of molecules, including deoxynucleotides, NAD and nucleotide sugars [92]. E-NPP3 is a member of the family of type II transmembrane proteins, which are composed of a short N-terminal cytoplasmic domain, followed by the transmembrane region, two somatomedin-like domains, the catalytic domain and a C-terminal endonuclease-like domain. CD203c is associated with malignant subversion and invasive properties [93]. CD203c has been defined as an activation-linked surface antigen on basophils and mast cells that is upregulated in response to IgE receptor cross-linking and is overexpressed on neoplastic mast cells in patients with systemic mastocytosis [94]. CD203c expression on basophils may implicate these cells in some allergic diseases [95].

CD30 is a member of the tumor necrosis factor receptor/nerve growth factor (TNFR/NGFR) superfamily [96]. Ligation of CD30 ligand (CD30L or CD153) to CD30 elicits multidirectional signals leading to either cell activation or apoptosis [96]. Under physiological conditions, expression of CD30 is restricted to T and B cells, mainly to activated Th2 cells. CD30 is expressed typically on the surface of Hodgkin’s Reed-Sternberg cells and anaplastic large cell lynphomas [96]. Human mast cells from normal donors do not express CD30. CD30 expression is upregulated aberrantly in most indolent and aggressive forms of systemic mastocytosis [97].

CD25 is α chain of IL-2 receptor. The high affinity IL-2 receptor (IL-2R) is a heterotrimer consisting of the IL-2R α chain (IL-2Rα, CD25) and the IL-2R β- and γ chains (IL-2Rβ and IL-2Rγ) [98]. CD25 serves as a major growth factor receptor by binding IL-2. The IL-2Rα does not contain an intracellular signaling domain, therefore binding to IL-2Rα alone does not result in T cell activation [98]. The high affinity IL-2R heterotrimer is expressed on activated T cells and regulatory T cells [98]. Mast cells from normal donors do not express CD25 during maturation and activation [99]. However, mast cells in systemic mastocytosis aberrantly display CD25, which is a diagnostic marker of neoplastic mast cells in systemic mastocytosis variants and in platelet-derived growth factor receptor alpha (PDGFRA)-associated myeloproliferative disorders [86].

Under allergic inflammatory conditions, “primed” mast cells produce IL-4 and IL-13 and express high levels of the FcεRI and the ligand for the surface antigen CD40, involved in T/B cell interactions leading to IgE production [100, 101]. IL-4 from mast cells can direct uncommitted helper T lymphocytes toward Th2 and also upregulate the FcεRI expression in mast cells [100]. Furthermore, locally synthesized IgE can upregulate the FcεRI expression on mast cells [102]. The augmented FcεRI on mast cells can bind an increased number of IgE-Ag complexes which in turn can enhance the sensitivity of mast cells to allergen resulting in the enhancement of the production of immunomodulatory cytokines /chemical mediators [103]. The upregulation of FcεRI is observed in mast cells from atopic asthma [104, 105]. By contrast, alveolar mast cells may lack expression of FcεRI in healthy subjects [106]. Supporting the emerging concept of alveolar inflammation in asthma, alveolar mast cells shift to a FcεRI-expressing phenotype in atopic and uncontrolled asthma [107]. Site-specific and disease-associated mast cell changes have also recently been described in most other inflammatory conditions of the lung.

Thus a robust upregulation of FcεRI-expression (and surface-bound IgE) on alveolar mast cells and nasal mast cells is a novel disease-specific feature of atopic asthma and AR. Changes in the alveolar mast cells in allergic asthma are associated with a Th2-skewed alveolar cell profile, which correlates significantly with the degree of clinical control [108, 109].

## Basophils

### Histamine and its metabolites as biomarkers of basophils

Histamine [2-(4-Imidazolyl)-ethylamine] is stored in the granules of basophils, as well as in mast cells, neurons and enterochromaffin-like (ECL) cells in the stomach. Platelets also contain histamine, and it can be produced also by certain bacteria, including those commensal in the upper respiratory tract. Oxidative deamination of histamine by diamine oxidase, yields ammonia, hydrogen peroxide, and imidazole acetaldehyde. Neither degradation product binds to one of the four G-protein coupled histamine receptors (HR1-4), and thus these products have no known pharmacological activities.

Due to its storage in secretory basophil granules and release during immunological activation, histamine can be considered as a biomarker of basophil activation in vitro. However, its practical usefulness as a basophil activation marker is limited by several issues. Technically, histamine assays are relatively laborious and expensive, as in the histamine ELISA detection methods, or require specialised equipment such as histamine autoanalyzers. Moreover, collecting plasma at an optimal time is challenging, because histamine undergoes first pass metabolism in the circulation, peaking about five minutes after onset of insect sting-initiated anaphylaxis and returning to baseline within 15 to 30 min. Also, plasma rather than serum is preferred for histamine measurements, because of concerns over basophil activation during blood clotting, and avoidance of small bore venipuncture needles and vacuum tubes is also recommended to decrease the likelihood of basophil shearing during blood collection. Further, traditional histamine measurements are bulk-type measurements which average the content or release of histamine across the studied cell population. Finally, while histamine is stored in large amounts in the granules of mature basophils (and mast cells), it is also secreted by haematopoietic progenitors [110] before being stored in the granules. Accordingly, it is also produced by other non-mast cell/non-basophil cells such as eosinophils, platelets, neutrophils, in addition to the aforementioned neurons, microglia and enterochromaffin-like cells. Thus, histamine elevations per se do not unequivocally identify the basophil as the source of the histamine. Increased histamine concentrations in the absence of concomitant rises in levels of other mast cell mediators, e.g., PGD2, can implicate infiltrating basophils as the primary source in allergen-induced late phase reactions [111]. Histamine released from blood cells will in most cases be of basophil origin, though assessment of basophil activation to allergens in vitro is more commonly carried out at present by measuring membrane expression of CD63 and/or CD203c by flow cytometry [112].

As an alternative to measurements of extracellular histamine or its metabolites, a HistaFlow technique using an enzyme-affinity-fluorochrome method using fluorescently labelled DAO to measure intracellular histamine [113], is able to capture data at the single cell level, has been used to monitor basophil responsiveness during immunotherapy [114], potentially serving as a surrogate biomarker for monitoring immunotherapy efficacy.

### LTC4 and its metabolites

Basophils rapidly synthesize LTC4 within minutes upon allergen binding to specific IgE, occupying high affinity receptors (FcεRI), as well as to other diverse stimuli [115, 116]. Phospholipids in basophils, including phosphatidylcholine and phosphatidylinositol, likely contribute to the arachidonic acid source from which LTC4 is then derived via the actions of calcium-sensitive 5-lipoxigenase. However, there are a variety of other cell types potentially contributing to the production of LTC4 in vivo, including mast cells, eosinophils and macrophages [117]. Nonetheless, its synthesis and secretion from washed leukocytes has long functioned as an in vitro/ex vivo assay to evaluate the allergic status, since basophils within these suspensions are the sole source of LTC4 following IgE/FcεRI-dependent activation [118]. The relative ease of measuring LTC4 in culture supernatants using commercially available enzymatic immunoassays (EIA) further enhances the utility of this mediator as a marker of basophil activation in vitro.

### Monoclonal antibody, J175-7D4, to pro-major basic protein 1

Studies of basophils [119] have been hampered both by the paucity of this cell type and by the difficulty of finding it in tissue. The monoclonal J175-7D4 antibody binds the propeptide region of bone marrow proteoglycan (gene name PRG2), but not the mature eosinophil granule major basic protein after removal of the propeptide [120]. Immunostaining with J175-7D4 is basophil-specific in human skin, nasal polyps, and esophagus. J175-7D4 does not stain mature mast cells or eosinophils; however, it does stain eosinophil precursors. Clone 2D7 antibody, commercially available, also recognizes a precursor form of eosinophil granule major basic protein, labelling only basophils, and has been used to identify basophils in tissues, including formalin-fixed, paraffin-embedded specimens [121], and in suspension by flow cytometry [122]. However, in our experience, 2D7 staining is inconsistent and can be weak in formalin-fixed paraffin-embedded tissue, and basophil staining is consistently brighter with J175-7D4 than with 2D7. In formalin-fixed, paraffin-embedded tissue, 7D4 antibody works well after basic EDTA antigen retrieval.

### Surface activation markers

Basophils are the least common of the classically described peripheral blood leukocyte populations. Because of their expression of FcεRI and easy accessibility in peripheral blood, basophils are often used as an experimental system to examine IgE mediated cell activation. Additionally, basophils are clearly an important allergic effector cell population in their own right [123].

Basophils can be physically separated from peripheral blood mononuclear cells and their production of soluble inflammatory mediators studied in bulk culture, in a manner similar to that noted above for mast cells. The ability to identify basophils using multicolor flow cytometry has facilitated their study in whole blood without such labor-intensive separation. In such work, basophils are typically identified by their expression of cell surface markers such as CD123, CCR3, or CRTH2 [124, 125], usually in combination with their low side-scatter properties [126].

The two best-described markers of human basophil activation are CD63 and CD203c [112, 124, 125]. In resting basophils, CD63 is located primarily in secretory granule membranes, and CD203c, reportedly in membranes of vesicles that are rapidly expressed after activation, are both translocated to the cell surface when basophils are activated to degranulate, e.g., after aggregation of FcεRI, where they can be detected by flow cytometry [89]. Bimodal surface expression of CD63 is typically detected on allergen-activated basophils in whole blood, whereas CD203c expression is seen on nearly all basophils regardless of their purity. Surface CD203c but not CD63 may be detected after activation that does not cause degranulation, e.g., after stimulation with IL-3 alone [127]. Thus, CD63 and CD203c are complementary for assessing basophil activation. In addition to CD63 and CD203c, multiple other granule membrane associated proteins, including CD13, CD107a, and CD164, exhibit similar upregulation [128]. These markers fall into two families that respectively represent anaphylactic and piecemeal degranulation processes [129].

### Basogranulin

Basogranulin was initially described as a high molecular weight granule protein that was a specific marker of basophils [130]. Basogranulin is secreted from granules after both IgE- and non-IgE mediated stimuli [131]. Basogranulin can be used as a specific immunohistochemical marker to identify basophils in tissue [132].

## Conclusions

Mast cells, eosinophils and basophils play a fundamental role in several inflammatory, allergic and autoimmune diseases and they contribute to defense against infectious organisms and to development of many types of cancer. The role of these cells in human diseases may be beneficial, for example in infectious diseases, or detrimental, as in allergic disorders. Thus, proper assessment of the degree of activation of these cells in vivo is desirable to monitor the course of disease and to assess the disease response to therapy. In this review, a number of molecules have been discussed that are biomarkers of the activation of mast cells, eosinophils and basophils. There is good evidence that these surrogate markers are useful in assessing the state of activation in vivo of mast cells, eosinophils and basophils in a variety of human diseases. In most cases these markers reflect the involvement of a given cell in a particular disease and their levels in blood or body fluids often correlate to the severity of the disease. Some of the markers are relatively cell-specific, e.g., cationic proteins for eosinophils and tryptase for mast cells. Others, such as histamine and cysteinyl leukotrienes, are not cell-specific.

In some cases the biomarkers are clearly reflective of their involvement in the pathogenesis of a disease. For example, histamine in allergic rhinitis and cysteinyl leukotrienes in asthma are both associated with pathology and respond to pharmacologic intervention. In the case of other markers, their role, if any, in the disease pathogenesis is still unclear. Surface markers and activation molecules expressed on the membrane of mast cells, eosinophils and basophils can, in some cases, be used for in vitro diagnosis of allergic diseases. Tryptase and ECP are considered useful diagnostic tools to measure mast cell and eosinophil involvement in mastocytosis and in hypereosinophilic syndromes. Many biomarkers of mast cell, eosinophil and basophil activation are modulated by pharmacologic treatments and, therefore, in some instances are used to monitor the response of disease to treatment.

While there are currently many good candidates as surrogate markers of mast cell, eosinophil and basophil activation in vivo, there is still work that must be done to fully establish the usefulness of these markers in clinical practice. First, the best techniques to measure these molecules should be employed and made more widely available for use in clinical studies and in the management of clinical disease. Second, we should better understand the significance of biomarkers of human inflammation and we should define how they correlate with specific clinical phenotypes. Finally, a better understanding of the mechanisms regulating the secretion and expression of biomarkers and their pharmacological modulation will have practical application in the management of diseases associated with mast cell, eosinophil and basophil activation.

## Abbreviations

ACE: angiotensin-converting enzyme
BAL: bronchoalveolar lavage
CA3: carboxypeptidase A3
CCR: c-c motif receptor
CD: cluster of differentiation
CEACAM: carcinoembryonic antigen-related cell adhesion molecule
CLC: Charcot-Leyden crystal protein
COX-1: cycloxygenase-1
CRTH2: chemoattractant receptor-homologous molecule expressed on th2 cells
Cyst LTs: cysteinyl leukotrienes
DCs: human dendritic cells
ECE: endothelin converting-enzyme
ECL: enterochromaffin-like cells
ECP: eosinophil cationic protein
EDN: eosinophil-derived neurotoxin
EIA: enzymatic immunoassays
EoE: eosinophilic eosophagitis
EPX: eosinophil peroxidase
Fc: fragment crystallizable (of immunoglobulin)
FENO: fraction of exhaled nitric oxide
FEV1: forced expiratory volume in 1 s
FVC: forced vital capacity
HPLC: high-performance liquid chromatography
ICAM: intercellular adhesion molecule
ICS: inhaled corticosteroid
IL: interleukin
ILA: induced by lymphocyte activation
KIT: stem cell factor receptor
LO: lipoxygenase
LT: leukotriene
mAb: monoclonal antibody
MBP: major basic protein
PAPP-A: pregnancy-associated plasma protein-A
PC20: provocative concentration of methacholine or histamine producing a 20 % fall in FEV_1_
PDGFRA: platelet-derived growth factor receptor alpha
PGD2: prostaglandin D2
PSGL: P-selectin glycoprotein ligand
R: receptor
RNase: ribonuclease
ROC: receiver-operating characteristic
SLPI: secretory leukocyte protease inhibitor
SNPs: single nucleotide polymorphisms
Tele-MIAA: 1-methyl-4-imidazole acetic acid
TNFR: tumor necrosis factor receptor
TNFRSF: tumor necrosis factor receptor superfamily member.
Tregs: human regulatory T cells
